# Association of Constipation with Modes of Delivery: A Retrospective Questionnaire-based Study

**DOI:** 10.1007/s00192-024-05824-1

**Published:** 2024-06-07

**Authors:** G. Marije Hierink, Lauret A. M. Brinkman, G. G. Alec Malmberg, Hugo W. F. van Eijndhoven, Monika Trzpis, Paul M. A. Broens, on behalf of the LIOMOC-study group, Tessa van Wijk

**Affiliations:** 1 Department of Surgery, Anorectal Physiology Laboratory, University of Groningen, University Medical Center Groningen, Hanzeplein 1, PO Box 30 001, 9700 RB Groningen, The Netherlands; 2 Department of Obstetrics and Gynecology, University of Groningen, University Medical Center Groningen, Groningen, The Netherlands; 3 Department of Obstetrics and Gynecology, Isala Hospital, Zwolle, The Netherlands; 4 Department of Geriatric Medicine, University of Groningen, University Medical Center Groningen, Groningen, The Netherlands; 5 Department of Surgery, Division of Pediatric Surgery, University of Groningen, University Medical Center Groningen, Groningen, The Netherlands

**Keywords:** Vaginal delivery, Cesarean section, Parous women, Multiparous, Pelvic floor disorders

## Abstract

**Introduction and Hypothesis:**

Pelvic floor damage can contribute to pelvic floor dysfunction, including constipation. Most studies focus on constipation during pregnancy, whereas information regarding the mode of delivery in relation to constipation is limited. We hypothesise that women with a history of vaginal delivery report constipation more often than women with a history of caesarean section.

**Methods:**

This was a retrospective cross-sectional multicentre study conducted in the Netherlands. All included patients (*n* = 2,643) completed the Groningen Defecation and Fecal Continence questionnaire to assess bowel problems of the last 6 months. Parametric tests, Chi-squared, univariable and multivariable regression analyses were performed.

**Results:**

Among 2,643 parous women, 2,248 delivered vaginally (85.1%) and 395 (14.9%) by caesarean section. Altogether, 649 women (24.6%) suffered from constipation. Women in the vaginal delivery group were constipated more often than women in the caesarean section group (25.5% versus 19.0%, *p* = 0.005). For women who had delivered vaginally, multivariable regression analysis showed an odds ratio for constipation of 1.47 (95% confidence interval, 1.109–1.938, *p* = 0.007). The odds ratio for constipation in women with a spontaneous perineal tear was 1.4 times higher than in women with an intact perineum (*p* = 0.030). Furthermore, the vaginal delivery group reported difficulties regarding bowel emptying (*p* = 0.048), straining (*p* = 0.027), incomplete defecation (*p* = 0.043), not able to defecate daily (*p* = 0.018), manually assisted defecation (*p* = 0.015) and had higher Renzi scores (*p* = 0.043) more often.

**Conclusions:**

Women in the vaginal delivery group have higher prevalences and odds ratios for constipation. Furthermore, a perineal tear during vaginal delivery increases the odds ratio for constipation.

**Supplementary information:**

The online version contains supplementary material available at 10.1007/s00192-024-05824-1

## Introduction

The postpartum period is associated with various gastrointestinal complaints, including constipation, which greatly impacts patients’ well-being and productivity and affects medical consultation costs [1]. A recent study showed that the prevalence of constipation in the Dutch female population is 29.3%, which includes both nulliparous and parous women [2]. Currently, conflicting data exist on the possible association between constipation and the mode of delivery [3]. Most obstetric studies focused on pelvic floor dysfunction in general, whereas comprehensive studies focusing on the prevalence and severity of constipation in relation to the mode of delivery are rare [4–6]. Previously, the prevalence of constipation following childbirth could not be studied owing to a lack of standardised, validated questionnaires, especially ones addressing the mode of delivery [7].

Women with constipation may suffer from straining, lumpy or hard stools, incomplete defecation, anorectal blockage and/or the need for manual manoeuvres to defecate [8]. Damage to the pelvic floor, which may occur during childbirth, can impair pelvic floor function and influence the defecation process, which could lead to inappropriate defecation habits. These, in turn, could contribute to the development of obstructed defecation syndrome. Obstructed defecation syndrome is a complicated stool passage and can be caused by difficulties in the relaxation of the pelvic floor or dyssynergic defecation, which can ultimately lead to the onset of constipation [9]. Therefore, it can be assumed that damage to the pelvic floor caused by childbirth can influence the onset of constipation [10]. Whether caesarean section (CS) protects against developing constipation is unknown [4, 7]. We hypothesise that women with a vaginal delivery might have a higher prevalence of constipation.

The primary aim of our study was to provide a comprehensive inventory of the prevalence of constipation in relation to the mode of delivery. Secondarily, we aimed to investigate whether other contributing obstetric factors influence the prevalence of constipation and obstructive defecation disorders.

## Materials and Methods

### Study Design and Data Collection

This was a retrospective, cross-sectional, questionnaire-based multicentre study. It was performed with the approval of the Medical Ethics Review Board of the University Medical Center Groningen (METc 2016.265/M16.195647) and Isala Hospital Zwolle, the Netherlands. The outcome of this study was to investigate the prevalence of constipation in relation to the modes of delivery and to investigate possible contributing obstetric factors.

**Fig. 1:**
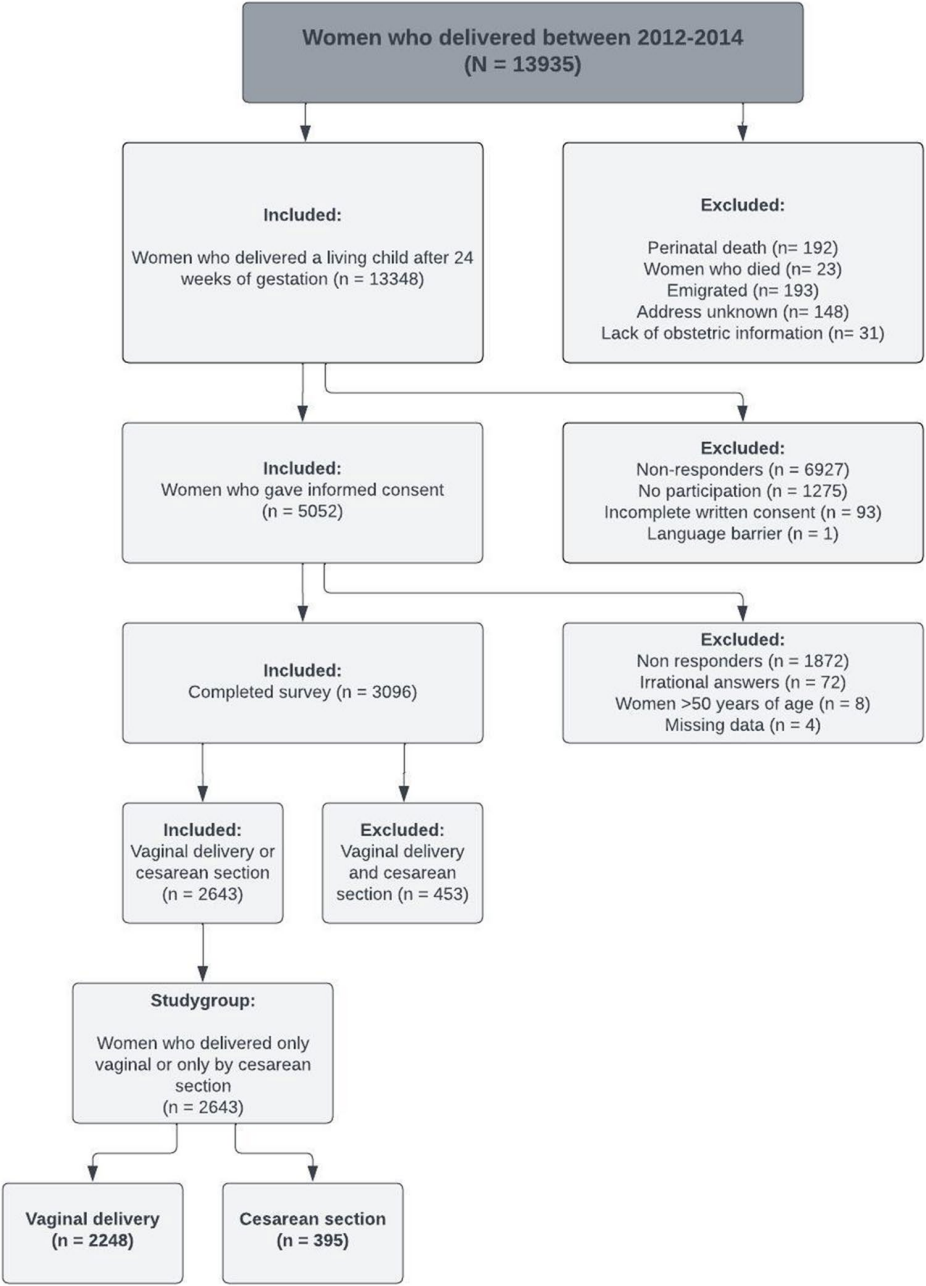
Flowchart of the study population

Women who had delivered a living child at least once between 2012 and 2014, either at University Medical Center Groningen or Isala Hospital, were invited between 2016 and 2018 to participate in this study (*n* = 13,348) to fill out the Groningen Defecation and Fecal Continence (DeFeC) questionnaire. We did not invite women who had a history of perinatal death (*n* = 192), women who had emigrated (*n* = 193), women whose address was unknown (*n* = 148), women on whom no obstetric information was available (*n* = 31) and women who had died between delivery and the onset of our study (*n* = 23). Furthermore, we excluded non-responders (*n* = 6,927), women who did not want to participate (*n* = 1,275), women who had incomplete written consent (*n* = 93) and women with a language barrier (*n* = 1). All women who gave their informed consent were sent a digital link that enabled them to complete the DeFeC questionnaire (*n* = 5,052; Fig. 1) [11].

The DeFeC questionnaire is self-administered and validated for the Dutch population regarding faecal and urinary problems that were present during the last 6 months of filling out the questionnaire (Supplementary file 1) [11]. The respondents received no compensation for completing the questionnaire. The questionnaire includes questions about faecal and urinary complaints of the last 6 months, allowing us to compute various problems according to the Rome IV criteria and to compute Renzi scores. It also contains questions regarding demographic information and obstetric history. Furthermore, the DeFeC questionnaire acquires information on respondents’ medical history regarding bowel or pelvic floor surgery and other factors that could have had a negative influence on defecation.

Women who failed to complete the questionnaire despite having given informed consent (*n* = 1,872), who were older than 50 years (*n* = 8) or whose answers were irrational (*n* = 72) were excluded. Data were missing on 4 women, which also excluded them from analysis. Finally, women who reported having both a vaginal delivery and a CS were excluded (*n* = 453). The remaining study group (*n* = 2,643) was divided for analysis into two subgroups: vaginal delivery only (*n* = 2,248) and CS only (*n* = 395).

### Definitions

We defined functional constipation according to the Rome IV criteria [8]. At least two of the following complaints should have been reported: straining, lumpy or hard stools, incomplete evacuation, anorectal blockage, manually assisted defecation, and reduced stool frequency (Supplementary file 2). Women who did not meet these requirements were defined as not constipated. Furthermore, the Renzi score was performed, which is a clinical severity index for obstructed defecation syndrome, with 0 as the minimum and 20 as the maximum score. A cut-off ≥ 9 of the Renzi score is used to differentiate between healthy patients and patients with obstructed defecation syndrome [12].

We defined parous women as women who had given birth at least once to an infant weighing 500 g or more. In multivariable analysis, we adjusted for diseases that could influence bowel function. This comprised women with a history of intestinal resection, intestinal or perianal fistula, anal sphincter or haemorrhoid surgery, women using a stoma, suffering inflammatory bowel diseases, diabetes mellitus, rectal prolapse, stroke, neurological abnormalities (e.g. multiple sclerosis and spinal cord injury), congenital abnormalities (e.g. anal atresia, anorectal malformation, Hirschsprung disease, spina bifida, sacrococcygeal syndrome and/or surgery for these abnormalities) [11].

### Statistical Analysis

To determine the distribution of continuous variables, we used a Q-Q plot. Parametric tests were used for normally distributed continuous variables and reported means ± SDs. We used Chi-squared or Fisher’s exact tests to analyse nominal and ordinal variables. Statistical significance was defined as a* p* ≤ 0.05, and we used odds ratios (ORs) with 95% confidence intervals (CIs) to represent the outcomes of univariable and multivariable regression analysis.

We used multivariable regression analyses to examine independent variables between constipation and obstetric or non-obstetric risk factors. In the multivariable analysis, we adjusted for age, body mass index, diseases that could influence bowel function, medication for other comorbidities, number of deliveries, the weight of the heaviest infant, duration of pushing, use of forceps or vacuum extraction, and the condition of the perineum.

The statistical analysis was performed using IBM Statistical Package for the Social Sciences version 28.0 software (IBM, Armonk, NY, USA). To display the results graphically, we used GraphPad Prism version 9.1.0 (GraphPad Software, San Diego, CA, USA). We used Lucidchart to display our flowchart.

## Results

### Respondents’ Characteristics

Table 1 contains the detailed characteristics of the respondents. Out of the total group of 2,643 women, 2,248 (85.1%) reported a history of vaginal delivery and 395 (14.9%) women reported a history of CS. The women in the vaginal delivery group were significantly younger than those in the CS group (35.81 versus 36.95, *p* = 0.001). The vaginal delivery group also had a lower body mass index than the CS group (24.82 versus 26.31, *p* = 0.001). Women who had given birth vaginally had significantly more children than women who had undergone a CS (*p* = 0.001). There were no differences in the use of medication related to bowel management between the two groups. However, in the CS group, 108 (27.6%) women used medicines for non-bowel-related comorbidities, compared with 501 women (22.4%) women in the vaginal delivery group (*p* = 0.026). There were no differences in both groups regarding diet habits related to constipation, such as daily fluid intake, fruit intake, vegetable intake and bread intake. Also, the uses of laxatives and enemas were comparable. Furthermore, women in the CS group had a disease that could influence defecation more often (16.5% versus 12.3%, *p* = 0.024), as specified in Table 1.

**Table 1:** Respondents’ characteristics

	Total	Vaginal delivery (*n* = 2,248)	Caesarean section (*n* = 395)	*p* value
Age (years)	35.98 (SD 4.63)	35.81 (SD 4.60)	36.95 (SD 4.67)	0.001
Body mass index (continuous)	25.04 (SD 4.93)	24.82 (SD 4.82)	26.31 (SD 5.38)	0.001
Number of deliveries				0.001
1	448 (17.0%)	317 (14.1%)	131 (33.2%)	
2	1,449 (54.8%)	1,235 (54.9%)	214 (54.2%)	
≥ 3	746 (28.2%)	696 (31.0%)	50 (12.7%)	
Use of medicine for defecation disorders				0.179
No	2,421 (91.6%)	2,070 (92.1%)	351 (88.9%)	
Laxatives	198 (7.5%)	159 (7.1%)	39 (9.9%)	
Anti-diarrhoea	23 (0.9%)	18 (0.8%)	5 (1.3%)	
Combination	1 (0.0%)	1 (0.0%)	0 (0.0%)	
Use of medicine for other comorbidities				0.026
No	2,020 (76.8%)	1,736 (77.6%)	284 (72.4%)	
Yes	609 (23.2%)	501 (22.4%)	108 (27.6%)	
Daily fluid intake				0.871
More than 1.5 l	1,804 (68.3%)	1,533 (68.2%)	271 (68.6%)	
Less than 1.5 l	839 (31.7%)	715 (31.8%)	124 (31.4%)	
Fruit intake				0.413
At least two pieces of fruit	1,875 (52%)	1,177 (52.4%)	198 (50.1%)	
Fewer than two pieces of fruit	1,268 (48.0%)	1,071 (47.6%)	197 (49.9%)	
Vegetable intake				0.883
At least 150 g	1,972 (74.6%)	1,676 (74.6%)	296 (74.9%)	
Less than 150 g	670 (25.4%)	571 (25.4%)	99 (25.1%)	
Slice of brown bread or whole grain bread				0.159
No	745 (28.2%)	622 (27.7%)	123 (31.1%)	
Yes	1,887 (71.8%)	1,625 (72.3%)	272 (68.9%)	
Use of laxatives				0.316
Never	2,449 (92.7%)	2,091 (93.0%)	358 (90.6%)	
Less than once a month	98 (3.7%)	80 (3.6%)	18 (4.6%)	
Monthly	39 (1.5%)	29 (1.3%)	10 (2.5%)	
Weekly	23 (0.9%)	19 (0.8%)	4 (1.0%)	
Daily	34 (1.3%)	29 (1.3%)	5 (1.3%)	
Usage of enema				0.286
No	2,616 (99.0%)	2,227 (99.1%)	389 (98.5%)	
Yes	27 (1.0%)	21 (0.9%)	6 (1.5%)	
Disease that could influence defecation				0.024
No	2,301 (87.1%)	1,971 (87.7%)	330 (83.5%)	
Yes	342 (12.9%)	277 (12.3%)	65 (16.5%)	

*SD* standard deviation

### Constipation-related Bowel Habits and Mode of Delivery

The Renzi score for obstructive defecation was significantly higher in the vaginal delivery group than in the caesarean group (*p* = 0.043). In the vaginal delivery group, the median score was 4 (minimum 0, maximum 16) compared with 3 (minimum 0, maximum 17) for women in the CS group (not presented in the table or figure).

**Fig. 2:**
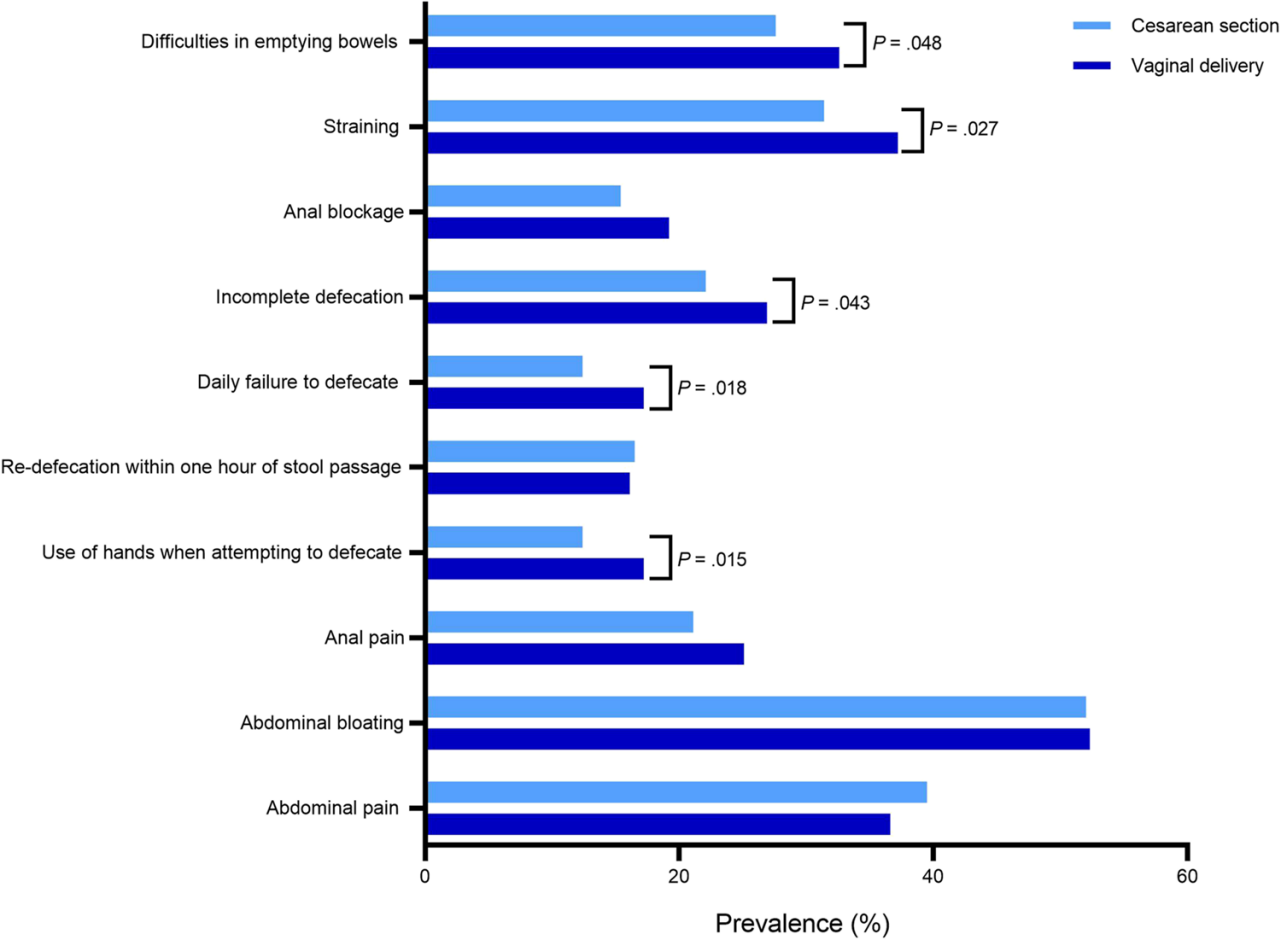
Bowel habits in women who gave birth by caesarean section or vaginal delivery

Women in the CS group reported fewer difficulties emptying their bowels (27.6% versus 32.6%, *p* = 0.048; Fig. 2). We noticed that the duration of difficulties emptying bowels and the average straining duration were comparable in both groups. Other bowel habits that were all significantly more common in the vaginal delivery group than in the CS group were straining (*p* = 0.027), feeling of incomplete defecation (*p* = 0.043), and not being able to defecate despite the urge to empty bowels daily (*p* = 0.018). Straining in particular was reported in 37.2% of the women in the vaginal delivery group, compared with 31.4% in the CS group (*p* = 0.027). With 26.9% versus 22.1% (*p* = 0.043), feeling of incomplete defecation was also more common in the vaginal delivery group. More often reported in the vaginal delivery group, but not significantly different, were anal pain (25.1% versus 21.1% *p* = 0.085) and anal blockage (19.2% versus 15.4%, *p* = 0.076). Women who had delivered vaginally reported manually assisted attempts to defecate more frequently than women with a history of CS (19.4% versus 14.2%, *p* = 0.015). In contrast, there was no significant difference in the prevalence of re-defecation within 1 h of stool passage, abdominal bloating or abdominal pain.

### Association Between the Prevalence of Constipation and Obstetric Parameters

We found that 649 (24.6%) women with a medical history of childbirth were constipated (Table 2). In this constipated group, 574 (25.5%) women had delivered vaginally versus 75 (19.0%) by CS. This difference between the two groups was statistically significant (*p* = 0.005).

**Table 2:** Obstetric factors and constipation in parous women

	Constipation			Univariable analysis		Multivariable analysis	
	No (*n* = 1,994)	Yes (*n* = 649)	*p* value	Odds ratio (95% CI)	*p* value		*p* value
Type of delivery^a^							
Only caesarean section	320 (81.0%)	75 (19.0%)	0.005	Reference	0.006	Reference	
Only vaginal delivery	1674 (74.5%)	574 (25.5%)		1.46 (1.12–1.91)		1.47 (1.11–1.94)	0.007
Age, years^b^	36.13 (SD 4.59)	35.51 (SD 4.74)	0.003	0.97 (0.95–0.99)	0.003	0.97 (0.95–0.99)	0.004
Body mass index, continuous^b^	25.00 (SD 5.04)	25.14 (SD 4.59)	0.552	1.01 (0.99–1.02)	0.552	1.00 (0.98–1.02)	0.751
Disease that could influence defecation^b^			0.138				
No	1747 (75.9%)	554 (24.1%)		Reference	0.138	Reference	
Yes	247 (72.2%)	95 (27.8%)		1.21 (0.94–1.57)		1.02 (0.76–1.36)	0.912
Medication for other comorbidities^b^			0.003				
No	1550 (76.7%)	470 (23.3%)		Reference		Reference	
Yes	431 (70.8%)	178 (29.2%)		1.36 (1.11–1.67)	0.003	1.39 (1.11–1.75)	0.005
Number of deliveries^b^			0.372				
1	327 (73.0%)	121 (27.0%)		Reference		Reference	
2	1105 (76.3%)	344 (23.7%)		0.84 (0.66–1.07)	0.160	0.85 (0.63–1.15)	0.283
≥ 3	562 (75.3%)	184 (24.7%)		0.89 (0.68–1.16)	0.369	0.92 (0.66–1.29)	0.624
Weight of heaviest infant, grams^b^	3639 (SD 635.97)	3673 (SD 604.37)	0.264	1.09 (0.93–1.27)	0.264	1.09 (0.92–1.29)	0.340
Duration of pushing^b^			0.348				
< 1 h	887 (75.2%)	292 (24.8%)		Reference		Reference	
1–2 h	537 (74.8%)	181 (25.2%)		1.02 (0.82–1.27)	0.829	1.01 (0.81–1.27)	0.918
> 2 h	247 (71.4%)	99 (28.6%)		1.29 (0.93–1.59)	0.150	1.16 (0.86–1.57)	0.323
Use of forceps or vacuum extraction^b^			0.264				
No	1212 (75.2%)	400 (24.8%)		Reference		Reference	
Yes	460 (72.9%)	171 (27.1%)		1.13 (0.91–1.39)	0.264	0.93 (0.73–1.19)	0.551
Condition of the perineum^b^			0.126				
Intact perineum	680 (76.8%)	205 (23.2%)		Reference		Reference	
Vaginal tear	207 (70.6%)	86 (29.4%)		1.38 (1.03–1.85)	0.034	1.39 (1.03–1.88)	0.030
Episiotomy	448 (74.2%)	156 (25.8%)		1.16 (0.91–1.47)	0.239	1.11 (0.85–1.45)	0.448
Combination	334 (72.6%)	126 (27.4%)		1.25 (0.97–1.62)	0.088	1.22 (0.92–1.61)	0.175

^a^Mode of delivery in the multivariable analysis was adjusted for age, body mass index, medication for other comorbidities, disease that could influence defecation and number of deliveries

^b^These variables were adjusted for age, body mass index, birthweight, duration of pushing, number of deliveries, condition of the perineum, use of forceps or vacuum extraction, diseases that could influence defecation and medication for other comorbidities

The vaginal delivery group had an increased odds ratio for constipation, as indicated by univariable analysis (OR, 1.46, 95% CI, 1.12–1.91, *p* = 0.006) and confirmed by multivariable regression analysis (OR, 1.47, 95% CI, 1.11–1.94, *p* = 0.007), which was corrected for age, body mass index, diseases that could influence defecation, medication for other comorbidities and number of deliveries. Of the constipated women with a history of vaginal delivery, 23.2% had an intact perineum, 29.4% had a spontaneous perineal tear, 25.8% had an episiotomy, and 27.4% had a combination of an episiotomy and a perineal tear. As shown in Table 2, the OR for constipation in women with a spontaneous perineal tear was 1.4 times higher than in women with an intact perineum. The OR for univariable analysis was 1.38 (95% CI, 1.03–1.85, *p* = 0.034) and for the multivariable analysis 1.39 (95% CI, 1.03–1.88, *p* = 0.030), which was adjusted for age, body mass index, birthweight, duration of pushing, number of deliveries, use of forceps or vacuum extraction, diseases that could influence defecation and medication for other comorbidities.

## Discussion

To the best of our knowledge, this study was the first to comprehensively investigate the prevalence and severity of constipation in relation to the mode of delivery with a validated defecation questionnaire [11]. We showed with multivariable analysis, in which we adjusted for cofounders contributing to constipation, that the prevalence of constipation was higher in women in the vaginal delivery group than in women in the CS group. Furthermore, we showed that the prevalence of specific defecation problems, such as difficulties emptying bowels, straining, feeling of incomplete defecation, not able to defecate daily despite the urge to empty bowels, and manually assisted attempts at defecating, was higher in women of the vaginal delivery group than in those in the CS group. Moreover, we found two additional factors associated with constipation: age and tearing of the perineum.

Our study showed that approximately a quarter of parous women experienced functional constipation, corresponding to previous studies [13, 14]. Mainly due to the different definitions of constipation, the documented prevalence of constipation in parous women ranges widely, from 2.6% to 26.9% [13]. The prevalence of constipation is higher in women than in men [15]. Obstetric injury of pelvic floor musculature and the anal sphincter complex after vaginal delivery may influence the onset of constipation and dyssynergic defecation [16, 17].

Dyssynergic defecation is the inability to coordinate the abdominal and pelvic floor muscles to evacuate stools, which could lead to constipation [18]. There is evidence that dyssynergic defecation, as determined by anorectal physiological testing, is the major pathophysiological derangement in constipation after vaginal delivery [16]. The study by Park and colleagues suggests that dyssynergic defecation might be associated with abnormal anal sphincter muscle function during vaginal delivery and seems to increase the risk of obstetric anal sphincter injuries [16]. Their study did not allow for a comparison between vaginal delivery and CS, possibly because of the small sample size [16]. One could suggest that women with a strong dyssynergic pelvic floor might have a less adaptive pelvic floor during labour and are more likely to be referred for CS. Shi and colleagues found that constipated pregnant women had a CS more often than non-constipated women but could not identify constipation as a risk factor for CS [19]. Our results, however, showed a lower prevalence of constipation in women in the CS group. Previously, pelvic floor dysfunction was shown to be associated with vaginal delivery, which partially explains our findings regarding constipation, even though most studies failed to distinguish between the types of pelvic floor dysfunction [3, 20].

In the vaginal delivery group, we found a higher prevalence of straining, a symptom that correlates with dyssynergic defecation. Besides pelvic floor injury due to childbirth, straining due to constipation can be harmful to the pelvic floor and can contribute to changes in anorectal physiology, which influences defecation habits [21]. Our study design, however, prevented us from knowing whether this was already present before childbirth or before the onset of constipation. Nevertheless, we also observed an increased prevalence of dyssynergic defecation symptoms in women from the vaginal delivery group, such as manually assisted defecation and inability to defecate daily despite the urge to empty bowels, which emphasised the extent of the constipation problem. Furthermore, we found that women in the vaginal delivery group have a significantly higher Renzi score, although not clinically relevant, as the scores were both below 9, which is the cut-off score for obstructed defecation syndrome [12]. This outcome was in line with that of another study [3]. It has also been reported that CS protects against pelvic floor dysfunction [20], except for faecal incontinence [3].

With regard to childbirth injuries causing dyssynergic defecation, Marchand and colleagues showed that anal sphincter tears occurred 2.94 times more often in women who reported dyssynergic defecation [18]. Interestingly, we found that a perineal tear was associated with constipation. However, we could not distinguish the extent of perineal tear in our questionnaire as it was a self-administered questionnaire. As far as we know, however, no studies demonstrated a relationship between constipation and perineal tears.

### Clinical Relevance

Constipation is one of the most commonly reported physical health problems after childbirth. The impact of constipation on the quality of life may not be underestimated as it has a major impact on patient’s well-being [1, 5, 22].

Surprisingly, existing research has predominantly focused on faecal incontinence rather than on constipation, even though the effect of constipation on the quality of life is larger [23]. Besides influencing the mother's physical health, it is known that constipation can also disturb the physical health of newborn infants and the attachment process between mothers and their infants [24]. Therefore, it is necessary to be aware of the risk of constipation and its consequences, not only during pregnancy but also after childbirth, especially as previous research conducted among a general Dutch population revealed that 49% of individuals experiencing constipation had never discussed their discomfort with anyone [2]. This underscores the prevailing social discomfort in addressing constipation openly, highlighting the possible undertreatment of constipation by patients and health care providers. Proactively addressing the issue and offering appropriate interventions, with special awareness to women with a history of a vaginal delivery, might prevent the worsening of neglected constipation.

With our study, we cannot conclude any causality between constipation and mode of delivery. However, this was an observational study, and we have shown a higher prevalence of constipation in the vaginal delivery group. Therefore, a prospective study would be invaluable to investigate the causality of constipation in relation to the mode of delivery and perineal trauma.

### Limitations and Strengths

We examined a Dutch hospital population, which possibly led to a selection bias towards women with higher-risk pregnancies. In the Netherlands, women with a medical indication give birth in a hospital under the supervision of a gynaecologist. Indications include complicated pregnancy or childbirth, wish for pain medication during childbirth by healthy women, the medical condition of the mother or whether the infant may be expected to require medical attention. We know that the CS rate of our study is lower than expected for the Dutch population. However, this can be explained owing to the exclusion of women with a history of both a vaginal delivery and a CS [25]. Currently, approximately 26.9% of Dutch women give birth at home or in the hospital with the help of a midwife [25]. In our study, all women had delivered in the hospital at least once, under the supervision of the gynaecologist. This implies that the health of a part of our sample might be impaired, which may bias our findings. However, the prevalence of constipation was 24.9% in our cohort, which is in accordance with other literature [2].

Another potential limitation is that the general analyses of obstetrical information were composed of the self-reports of participating women, which could lead to memory bias. The medical information was anonymised for the researchers. Consequently, more detailed medical information, such as the degree of perineal tears, could not be obtained. There is, however, sufficient evidence that medical reports corresponded with women’s own reports [26]. Therefore, we believe that the subjective self-reports of the parous women apply to this study. Furthermore, self-reporting is consistent and valid if symptoms are still present at the time of answering the questionnaire [27]. Also, it is unknown whether the higher prevalence of constipation resulted from vaginal delivery, because the situation before childbirth was unknown to us. Finally, the usage of forceps is rare in the Netherlands, and therefore, we did not distinguish between a forceps and a vacuum in our article.

The main strength of this study is the large sample size. To the best of our knowledge, this was the first study to examine the prevalence and severity of constipation in a vaginal delivery group and a CS group using a validated questionnaire. In addition, the DeFeC questionnaire is validated and extensively qualified, and contains questions regarding faecal and bowel function-related symptoms [11]. This allows us to determine functional constipation and other relevant, in this case, gynaecological, medical history [11]. This enabled us to adjust for bowel function-related diseases in our multivariable analysis, which could influence bowel function. Finally, the questionnaire is self administered, possibly reducing the embarrassment the respondents may have felt regarding their faecal health.

## Conclusion

In conclusion, our study showed that women in the vaginal delivery group, especially those with a vaginal tear of the perineum, have higher prevalences and odds ratios for constipation than women in the CS group. This presents an important message to all caregivers to alert women to the presence of constipation. Awareness of the problems may help women who are already prone to constipation to prevent them from developing more severe and chronic forms of constipation. It is important that more attention is given to constipation problems, not only during pregnancy but also postpartum, as constipation may have a major impact on patients’ quality of life [23].

### Supplementary Information

Below is the link to the electronic supplementary material.

Supplementary file1 (PDF 309 KB)

Supplementary file2 (DOCX 16 KB)

## Abbreviations

BMI: Body mass index
CI: Confidence interval
DeFeC: Defecation and Fecal Continence questionnaire
OR: Odds ratio
