# Supplementary material for: Association of Constipation with Modes of Delivery: A Retrospective Questionnaire-based Study
Source: Int Urogynecol J. 2024 Jun 7;35(7):1477–85. doi: 10.1007/s00192-024-05824-1 (PMC11315744; doi:10.1007/s00192-024-05824-1)
Supplement: Supplementary file 2 — Supplementary file2 (DOCX 16 KB) [file 192_2024_5824_MOESM2_ESM.docx]

| Question in DeFeC-questionnaire: | Answer |
| --- | --- |
|  |  |
| Functional constipation | *At least two of the following complaints according to the Rome IV criteria* |
| *1.1 On average, how often do you empty your bowels?* | *Twice a week or less* |
| *1.2 Bristol Stool chart* | *Separate hard lumps, sausage-shaped but lumpy or like a sausage but with cracks on its surface* |
| *2.2 How often did you have to strain hard to empty your bowel?* | *Several times a month or more* |
| *2.4 How often did you have trouble passing stools because it felt as if there was a blockage?* | *Several times a month or more* |
| *2.5 How often did it feel as if you had not completely emptied your bowels after passing stools?* | *Several times a month or more* |
| *3.9 Do you ever use your fingers or hands to help pass stools? (one or more)* | *Yes, I use my finger to press between my buttocks, just in front of the anus.*  *Yes, I use my finger to press between my buttocks, just behind the anus.*  *Yes, I use my fingers to remove stools from my anus.* |
|  |  |
| Obstructed defecation syndrome | *Points of Renzi score* |
| *q2.2 How often did you have to strain hard to empty your bowels?* | *0: Never*  *1: Less than once a month*  *2: Several times a month*  *3:Several times a week*  *4: Every day* |
| *Q2.5 How often did it feel as if you had not completely emptied your bowels after passing stools?* | *0: Never*  *1: Less than once a month*  *2: Several times a month*  *3: Several times a week*  *4: Every day* |
| *Q3.5 How often do you take laxatives to soften your stools/make it easier to empty your bowels?* | *0: Never*  *1: Less than once a month*  *2: Several times a month*  *3: Several times a week*  *4: Once a day*  *4: Several times a day* |
| *q3.7(1) Do you use an enema (= injecting a small amount of a medicine into the anus) to help pass stools?* | *1: Less than once a month*  *2: Several times a month*  *3: Several times a week*  *4: Once a day*  *4: Several times a day* |
| *q3.9(1) Do you ever use your fingers or hands to help pass stools? If so, how often do you use your fingers or hands when passing stools?* | *1: Less than once a month*  *2: Several times a month*  *3: Several times a week*  *4: Every day* |
| *Q2.10 How often did you have abdominal pain or cramps* | *0: Never*  *1: Less than once a month*  *2: Several times a month*  *3: Several times a week*  *4: Every day* |
